# Brain-derived neurotrophic factor is regulated via MyD88/NF-κB signaling in experimental *Streptococcus pneumoniae* meningitis

**DOI:** 10.1038/s41598-017-03861-z

**Published:** 2017-06-14

**Authors:** Danfeng Xu, Di Lian, Zhijie Zhang, Ying Liu, Jiaming Sun, Ling Li

**Affiliations:** 1 Department of Pediatric Neurology, Xinhua Hospital Affiliated to Shanghai Jiaotong University School of Medicine, Shanghai, 200092 P.R. China; 2 Department of Clinical Laboratory, Xinhua Hospital Affiliated to Shanghai Jiaotong University School of Medicine, Shanghai, 200092 P.R. China; 3 Department of Pathology, Xinhua Hospital Affiliated to Shanghai Jiaotong University School of Medicine, Shanghai, 200092 P.R. China

## Abstract

*Streptococcus pneumoniae* meningitis is an intractable disease of the central nervous system (CNS). Brain-derived neurotrophic factor (BDNF) is a member of the neurotrophic family and found to participate in the immune inflammatory response. In this study, we investigated if activation of the classical inflammatory signaling pathway, myeloid differentiation factor 88 (MyD88)/nuclear factor-kappa B (NF-κB), regulates BDNF expression in experimental *S. pneumoniae* meningitis. MyD88 knockout (*myd88*−/−) mice and wild-type littermates were infected intracisternally with *S. pneumoniae* suspension. Twenty-four hours after inoculation, histopathology of brains was evaluated. Cytokine and chemokine in brains and spleens was analyzed using ELISA. NF-κB activation was evaluated using EMSA. Cortical and hippocampal BDNF was assessed using RT-PCR and ELISA, respectively. BDNF promoter activity was evaluated using ChIP-PCR. *myd88*−/− mice showed an obviously weakened inflammatory host response. This diminished inflammation was consistent with worse clinical parameters, neuron injury, and apoptosis. Deficiency in MyD88 was associated with decreased BDNF expression. Furthermore, we identified a valid κB-binding site in the BDNF promoter, consistent with activation of NF-κB induced by inflammation. To sum up, MyD88/NF-κB signaling has a crucial role in up-regulating BDNF, which might provide potential therapeutic targets for *S. pneumoniae* meningitis.

## Introduction

*Streptococcus pneumoniae* meningitis is an invasive and often intractable disease of the central nervous system (CNS). Despite effective antibiotics and application of vaccinations, such infection is still associated with an unacceptably high morbidity and mortality^1^. The main limitation to advance in prevention and treatment of the disease is incomplete knowledge of its pathogenesis and pathophysiology. Generally, the host immune response, such as the activation of macrophages, production of cytokines and chemokines, and migration of leukocytes, is believed to be the first line of defense in response to bacterial invasion during the process of *S. pneumoniae* meningitis^2^. Toll-like receptors (TLRs), which are widely expressed in central resident macrophages, sense antigens from microorganisms, leading to the recruitment of myeloid differentiation factor 88 (MyD88) and the activation of downstream signaling pathways^3, 4^. MyD88 is crucial for the induction of a full innate inflammation response to most TLRs ligands, with the exception of TLR3^5^. Furthermore, the MyD88-dependent pathway elicits nuclear factor-kappa B (NF-κB) and mitogen-activated protein kinase (MAPK) activation, which drives robust gene expression of cytokines and pro-inflammatory mediators^6^. However, increasing evidence has demonstrated that activation of NF-κB can lead to uncontrolled expression of those pro-inflammatory mediators, which contributes to the pathogenesis of disease processes^7^. Innate immune response is now widely recognized as a double-edged blade possessing both protective and damaging properties^8^. There is now solid evidence that intense inflammatory host response causes important damage to the brain, thus inducing unfavorable outcomes of meningitis^9, 10^.

Brain-derived neurotrophic factor (BDNF) is a member of the neurotrophic family and is widely expressed in the adult brain. In CNS, multiple cell types express BDNF including neurons and glia^11^. BDNF promotes neuronal survival, maturation, and growth by binding to its high-affinity tropomyosin-related kinase receptor, type B (TrkB)^12, 13^. Dysfunction in the regulation of BDNF is associated with numerous disorders of CNS, including Alzheimer’s disease (AD), multiple sclerosis (MS), depression, and unacceptable outcomes of bacterial meningitis^14–17^. Our previous study showed that increased expression of BDNF following the acute *S. pneumonia* meningitis was alleviated after antibiotic treatment^18^. Furthermore, Barichello *et al*.^19^ demonstrated that down-regulated BDNF expression in the hippocampus was associated with cognition and memory deficiency in experimental *S. pneumoniae* meningitis. Interestingly, administration of exogenous BDNF increased the neuronal population in both the cortex and hippocampus, and reversed brain damage^20^. These findings indicate that regulatory expression of BDNF may be a part of the host inflammatory response in *S. pneumoniae* meningitis. However, the underlying regulatory mechanism is still not clear.

A recent report has shown that TLR agonists up-regulate nerve growth factor (NGF) in human intervertebral discs by activating and translocating NF-κB into the nucleus^21^. A tissue engineering study showed that hyaluronic acid-based hydrogels could attenuate inflammatory receptor activity in an interleukin (IL)-1β-induced inflammation model of nucleus pulposus cells, with down-regulation of NGF and BDNF^22^. Pro-inflammatory factors including endotoxins, cytokines, and oxidative stress have been reported to up-regulate BDNF in immune cells *in vitro*. In particular, tumor necrosis factor-alpha (TNF-α) has been demonstrated to increase BDNF expression via the extracellular signal-regulated kinase (ERK)/mitogen-activated protein kinase (MAPK) pathway in primary astrocytes^23^.

In this study, we aimed to investigate if activation of the classical inflammatory signaling pathway, namely the MyD88/NF-κB signaling pathway, regulates BDNF expression in experimental *S. pneumoniae* meningitis.

## Results

### Effect of MyD88 deficiency on characteristics of the meningitis and histopathology

**Figure 1:**
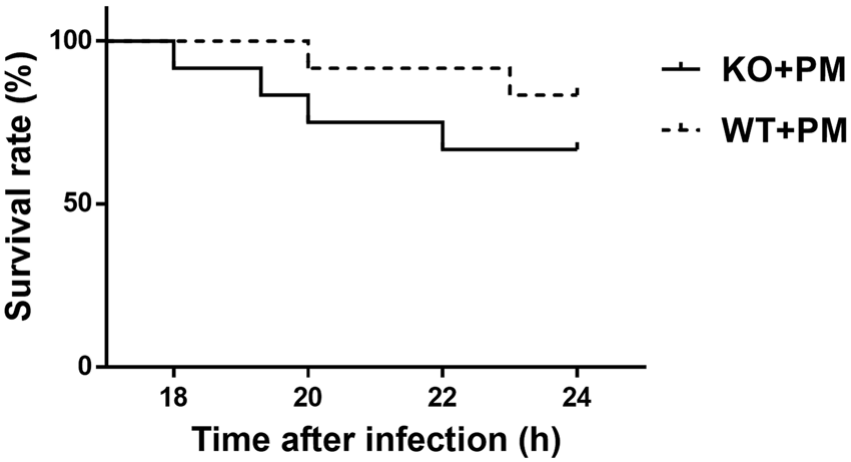
Kaplan-Meier curves showing the survival rates of mice with PM. Survival rate of *myd88*−/− and wild-type mice following *S. pneumoniae* infection were 66.7% and 83.3%, respectively. KO: knockout, PM: pneumoniae meningitis, WT: wild-type.

As a result of disease progression following inoculation with *S. pneumoniae*, all infected mice began to exhibit clinical symptoms of meningitis around 18 h after intracisternal injection, with loss of weight, hypothermia, lags in response and lethargy. Furthermore, the mortality rate was high during the acute disease phase, between 18 and 24 h after infection (Fig. 1). Of the 12 infected wild-type mice, 2 (16.70%) died within 24 h after inoculation, while 4 of 12 (33.3%) infected *myd88*−/− mice died during the same period. The survival rate of infected *myd88*−/− mice tended to be lower than infected wild-type mice; however, there was no significant variance (p > 0.05; log rank test). Data from two-way ANOVA indicated significant interactions between the variables (MyD88 and meningitis) on weight loss [F (1,30) = 8.687, p < 0.01] and clinical scores [F (1,36) = 5.345, p = 0.027]. In addition, infected *myd88*−/− mice lost more weight and had markedly enhanced clinical scores for severity of the disease compared to the infected wild-type mice (Table 1). None of the control mice induced with pyrogen-free saline showed symptoms of infection within the same observation period. All of the infected mice had positive bacterial cultures of the cerebellar homogenates but no pneumococci grew from the brains of uninfected mice.

**Table 1:** Weight loss and clinical scores in different groups (mean ± SD).

Groups	Weight loss (g)	Clinical score	Mortality rate
Infected myd88−/−	−2.83 ± 0.40^a,b^	3.33 ± 0.49^a,b^	33.30%
Control myd88−/−	−0.30 ± 0.30	0.00 ± 0.00	0
Infected wild-type	−2.03 ± 0.40^c^	2.42 ± 1.00^c^	16.70%
Control wild-type	−0.18 ± 0.27	0.00 ± 0.00	0

^a^p < 0.01, compared with control myd88−/−; ^b^p < 0.01, compared with infected wild-type; ^c^p < 0.01, compared with control wild-type. SD: standard deviation.

**Figure 2:**
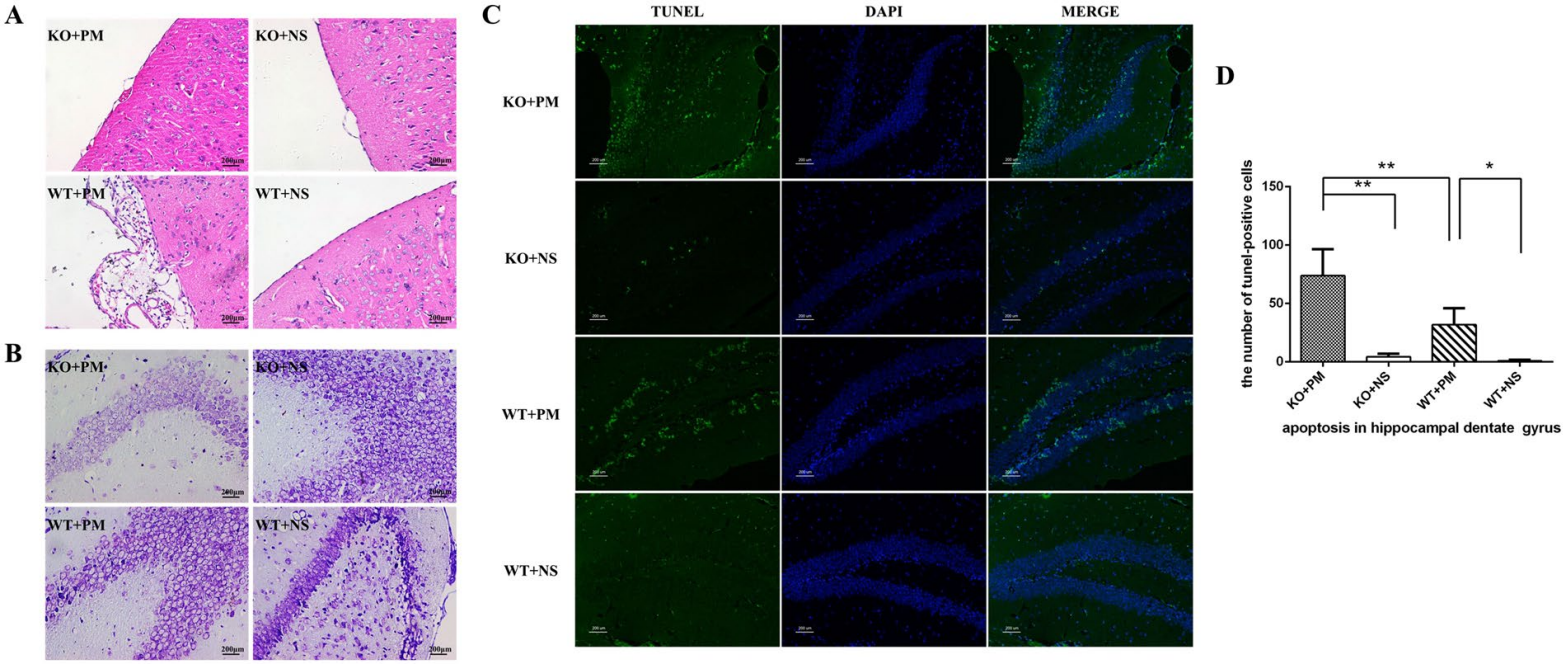
Effect of MyD88 deficiency on histological changes of mouse brains at 24 h after inoculation with *S. pneumoniae*. (**A**) H&E staining: Infected *myd88*−/− mice showed slight neutrophile granulocyte exudate in the subarachnoid cavity, while infected wild-type mice showed vast inflammatory exudate in the subarachnoid cavity and ventricle, with hemorrhage-like spots in the parenchyma. Control groups had no pathological changes of meningitis, with a smooth meninx. (**B**) Nissl staining: In infected *myd88*−/− mice group, the structure of most neurons in the hippocampus is incomplete, and Nissl staining is not uniform in the cytoplasm. In infected wild-type mice group, neuron loss is less severe than in infected *myd88*−/− mice. Nissl staining in control groups had no difference. (**C**) TUNEL immunofluorescence: There are more TUNEL-positive cells in infected *myd88*−/− mice than in infected wild-type mice. Few TUNEL-positive cells were observed in both control groups. (**D**) Quantitative analysis of the TUNEL results, *p < 0.05, **p < 0.01. KO: knockout, PM: pneumoniae meningitis, WT: wild-type, NS: normal saline.

Representative examples of H&E-stained brain sections of each group are shown in Fig. 2A. Infected wild-type mice showed vast inflammatory exudate in the subarachnoid cavity and ventricle, with hemorrhage-like spots in the parenchyma. However, the brains of infected *myd88*−/− mice showed obviously weak inflammatory response in the subarachnoid space. This finding corroborated with the dramatic weak antibacterial properties of *myd88*−/− mice to intracerebral *S. pneumoniae* infection.

In addition, neuronal injury in hippocampus was analyzed by using Nissl staining. As shown in Fig. 2B, neuron loss was more remarkable in the hippocampus of infected *myd88*−/− mice than in infected wild-type mice, with a more severely incomplete structure of neurons induced by intracerebral *S. pneumoniae* infection.

Furthermore, hippocampal apoptosis was investigated by TUNEL staining. Two-way ANOVA indicated significant interactions between the variables (MyD88 and meningitis) on hippocampal apoptosis [F (1,12) = 8.089, p = 0.015]. *S. pneumoniae* infection caused obvious apoptosis in the hippocampal dentate gyrus as compared with the control groups (Fig. 2C), and the number of TUNEL-positive cells was significantly higher in infected *myd88*−/− mice than in infected wild-type mice (Fig. 2D).

### Effect of MyD88 deficiency on inflammatory mediator production in brains and spleens

**Figure 3:**
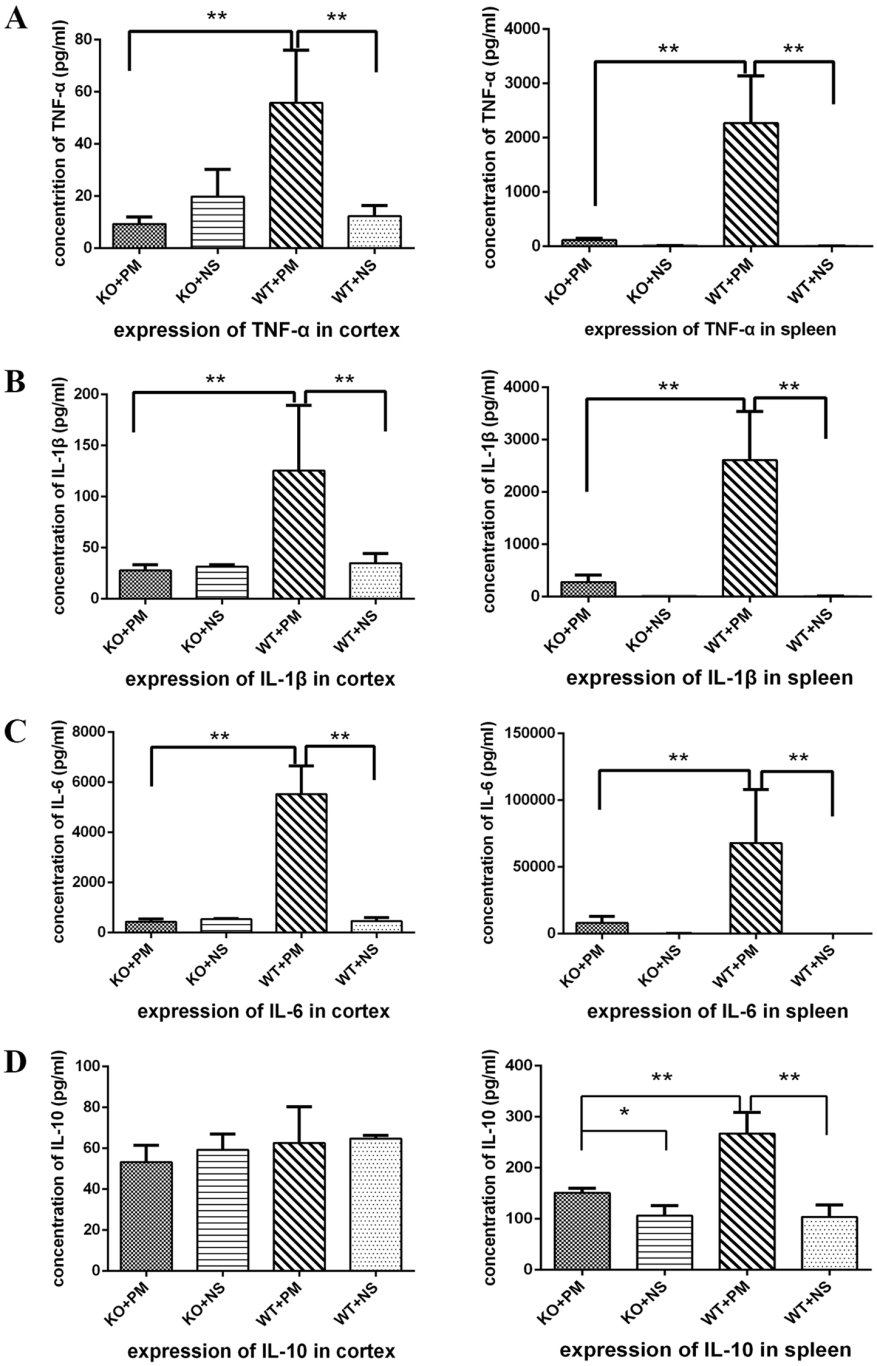
Effect of MyD88 deficiency on inflammatory mediator production in brains and spleens. *myd88*−/− and wild-type mice received intracisternal injection of *S. pneumoniae*, and cytokine/chemokine expression in brains and spleens were evaluated using ELISA kits for (**A**) TNF-α, (**B**) IL-1β, (**C**) IL-6, and (**D**) IL-10. In cortex and spleen homogenates from infected *myd88*−/− mice, no significantly increased expression of TNF-α, IL-1β, and IL-6 was detected. Inoculation with *S. pneumoniae* caused a significant increase in the expression of these inflammatory mediators in brains and spleens obtained from infected wild-type mice at 24 h after inoculation. Inoculation with *S. pneumoniae* led to increase of IL-10 in spleens both from infected *myd88*−/− mice and infected wild-type mice at 24 h after inoculation. Moreover, the expression of this anti-inflammatory mediator was much higher in infected wild-type mice than in infected *myd88*−/− mice. *p < 0.05, **p < 0.01. KO: knockout, PM: pneumoniae meningitis, WT: wild-type, NS: normal saline.

At 24 h after infection, the expression of cytokines and chemokines (i.e., TNF-α, IL-1β, IL-6, and IL-10) was evaluated by using ELISA. Two-way ANOVA indicated interactions significant between the variables (MyD88 and meningitis) on these inflammatory mediator productions (except expression of IL-10 in cortex). Data from two-way ANOVA for interaction on TNF-α in cortex and spleens is [F (1,22) = 29.185, p < 0.01] and [F (1,27) = 38.852, p < 0.01], respectively. Interactions between the variables on IL-1β is [F (1,20) = 11.845, p < 0.01] and [F (1,24) = 36.218, p < 0.01], respectively in cortex and spleens. Data of interactions on IL-6 is [F (1,20) = 115.165, p < 0.01] and [F (1,28) = 13.383, p < 0.01]. In the cortex, a two-way ANOVA of IL-10 expression indicated that there was no significant effect of the MyD88 group [F (1,18) = 2.244, p = 0.151], and there was no significant effect of the meningitis group [F (1,18) = 0.669, p = 0.424] with no significant interaction [F (1,18) = 0.149, p = 0.704]. However, in the spleens, interactions were identified between MyD88 and meningitis [F (1,25) = 35.366, p < 0.01]. *Streptococcus pneumoniae* infection led to massive cytokine and chemokine increase in both the cerebral cortex and spleen homogenates of wild-type mice (Fig. 3, except expression of IL-10 in cortex). In contrast, the expression of TNF-α, IL1-β, IL-6, and IL-10 was significantly attenuated in infected *myd88*−/− mice at the same time point, reflecting impaired immune activation.

### MyD88 is a major contributor to activation of NF-κB following *S. pneumoniae* administration

**Figure 4:**
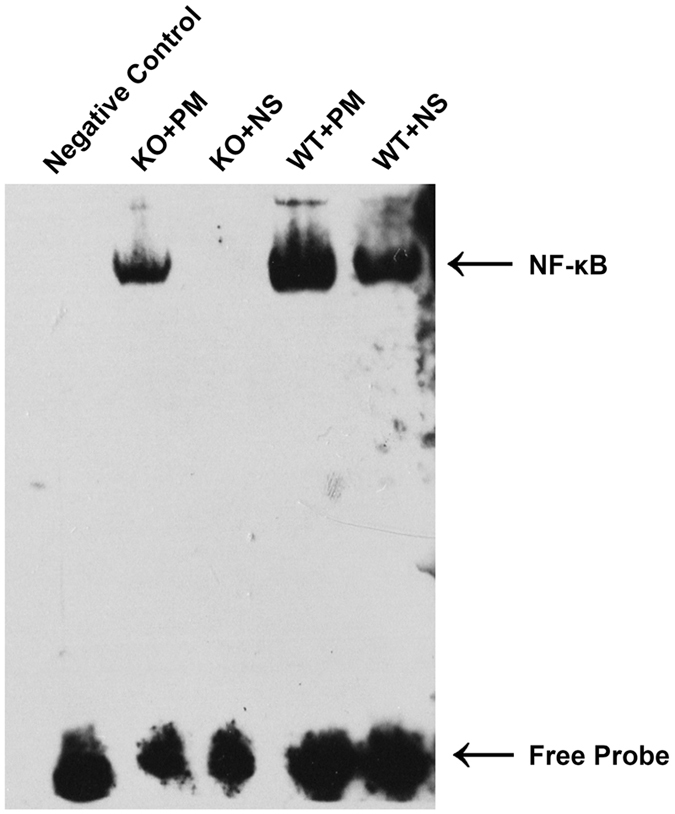
Effect of MyD88 deficiency on activation of NF-κB after inoculation with *S. pneumoniae*. NF-κB binding activity in the cortex in different groups was assessed by EMSA. Bacteria-induced NF-κB binding was markedly enhanced in infected wild-type mice compared to infected *myd88*−/− mice. KO: knockout, PM: pneumoniae meningitis, WT: wild-type, NS: normal saline.

To further determine the role played by MyD88 in *S. pneumoniae*-induced activation of NF-κB, EMSA was performed in nuclear extracts from brains derived from both *myd88*−/− and wild-type mice. NF-κB binding activation was significantly enhanced in infected wild-type mice compared with wild-type control mice (Fig. 4). However, this bacteria-induced NF-κB binding activation was markedly attenuated in *myd88*−/− mice. These data are in agreement with published studies^24, 25^ and indicate that MyD88 is required for *S. pneumoniae*-induced NF-κB binding activation in the CNS.

### MyD88 is required for *S. pneumoniae*-mediated increase in BDNF expression

**Figure 5:**
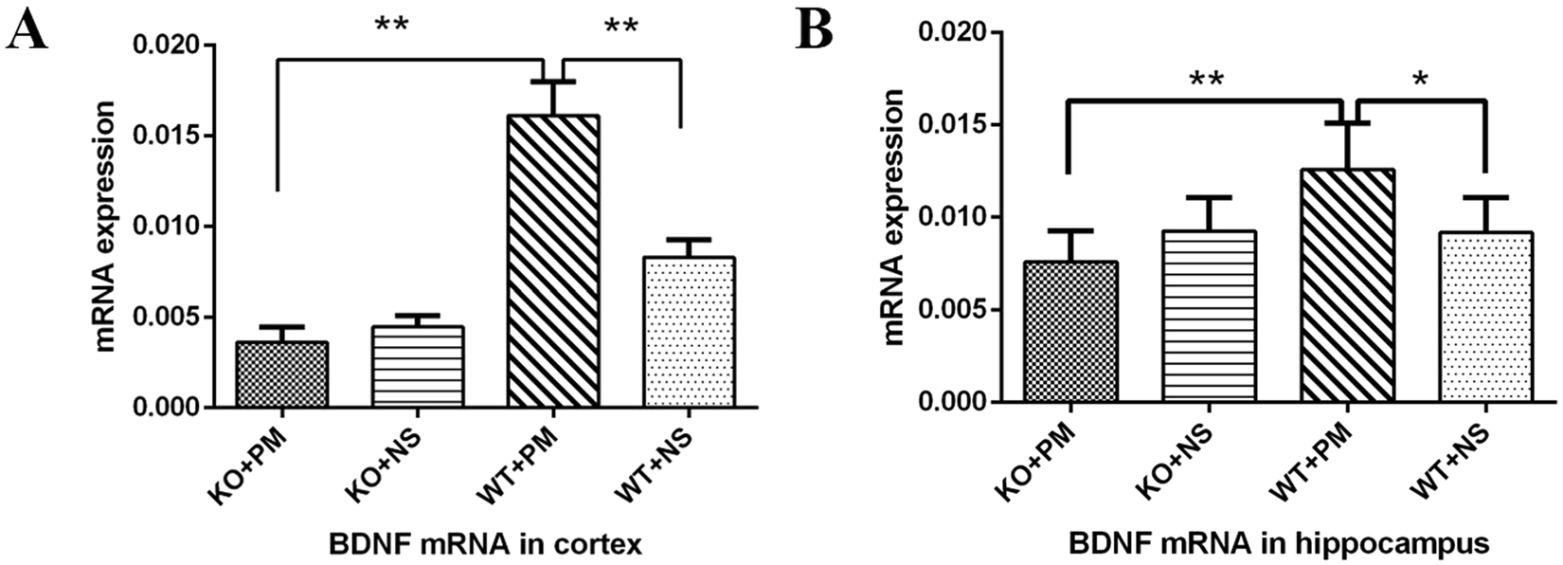
BDNF mRNA expression in the cortex and hippocampus at 24 h after inoculation with *S. pneumoniae*. BDNF mRNA expression in (**A**) the cortex and (**B**) hippocampus was significantly higher in infected wild-type mice than in control wild-type mice and infected *myd88*−/− mice. However, BDNF mRNA expression was similar between infected *myd88*−/− mice and control mice. *p < 0.05, **p < 0.01. BDNF: brain-derived neurotrophic factor, KO: knockout, PM: pneumoniae meningitis, WT: wild-type, NS: normal saline.

**Figure 6:**
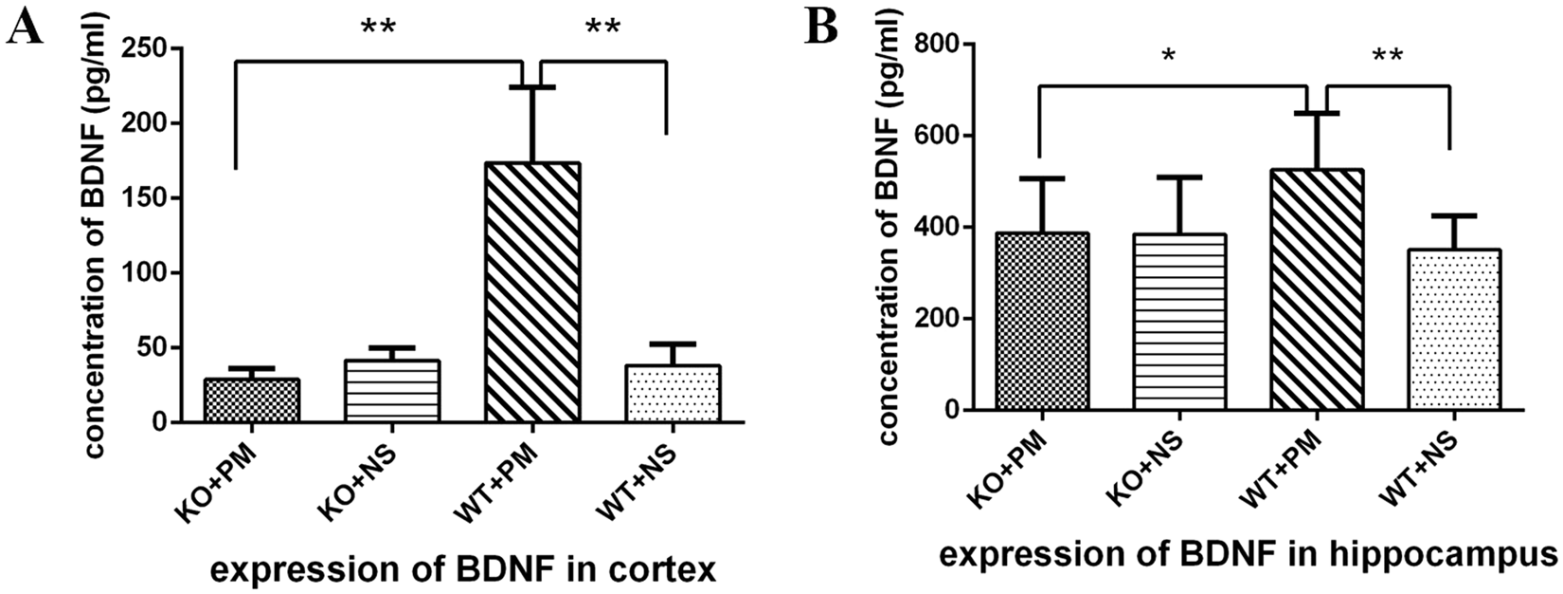
BDNF protein levels in the cortex and hippocampus at 24 h after inoculation with *S. pneumoniae*. BDNF in the cortex (**A**) and hippocampus (**B**) were significantly higher in infected wild-type mice than in control wild-type mice and infected *myd88*−/− mice. *p < 0.05, **p < 0.01. BDNF: brain-derived neurotrophic factor, KO: knockout, PM: pneumoniae meningitis, WT: wild-type, NS: normal saline.

Our previous studies have demonstrated that BDNF increases both in the cortex and hippocampus during the acute phase of *S. pneumoniae* meningitis, but decreases after antibiotics treatment^18^. In the present study, we further explored if MyD88 activation could induce the expression of BDNF in *S. pneumoniae* meningitis. At 24 h following inoculation, cortical cortex and hippocampus homogenates were collected and analyzed for expression of BDNF at the transcriptional and translational level. Data from two-way ANOVA indicated significant interactions between MyD88 and meningitis on BDNF expression. For BDNF mRNA [F (1,14) = 64.356, p < 0.01] and [F (1,20) = 9.731, p < 0.01], respectively in cortex and hippocampus. For BDNF protein [F (1,21) = 48.726, p < 0.01] and [F (1,17) = 6.902, p = 0.018], respectively in cortex and hippocampus. Infected wild-type mice promoted a strong trend of increased BDNF mRNA expression compared to saline controls (Fig. 5). Importantly, this bacteria-induced BDNF mRNA elevation was markedly attenuated in the cortical cortex and hippocampus isolated from *myd88*−/− mice. Next, we analyzed the protein level of BDNF in the cortical cortex and hippocampus. Similarly, we found that increases in levels of BDNF protein observed at 24 h following *S. pneumoniae* administration were absent in *myd88*−/− mice (Fig. 6).

### NF-κB directly acts with BDNF promoter expression in BV-2 strain

**Figure 7:**
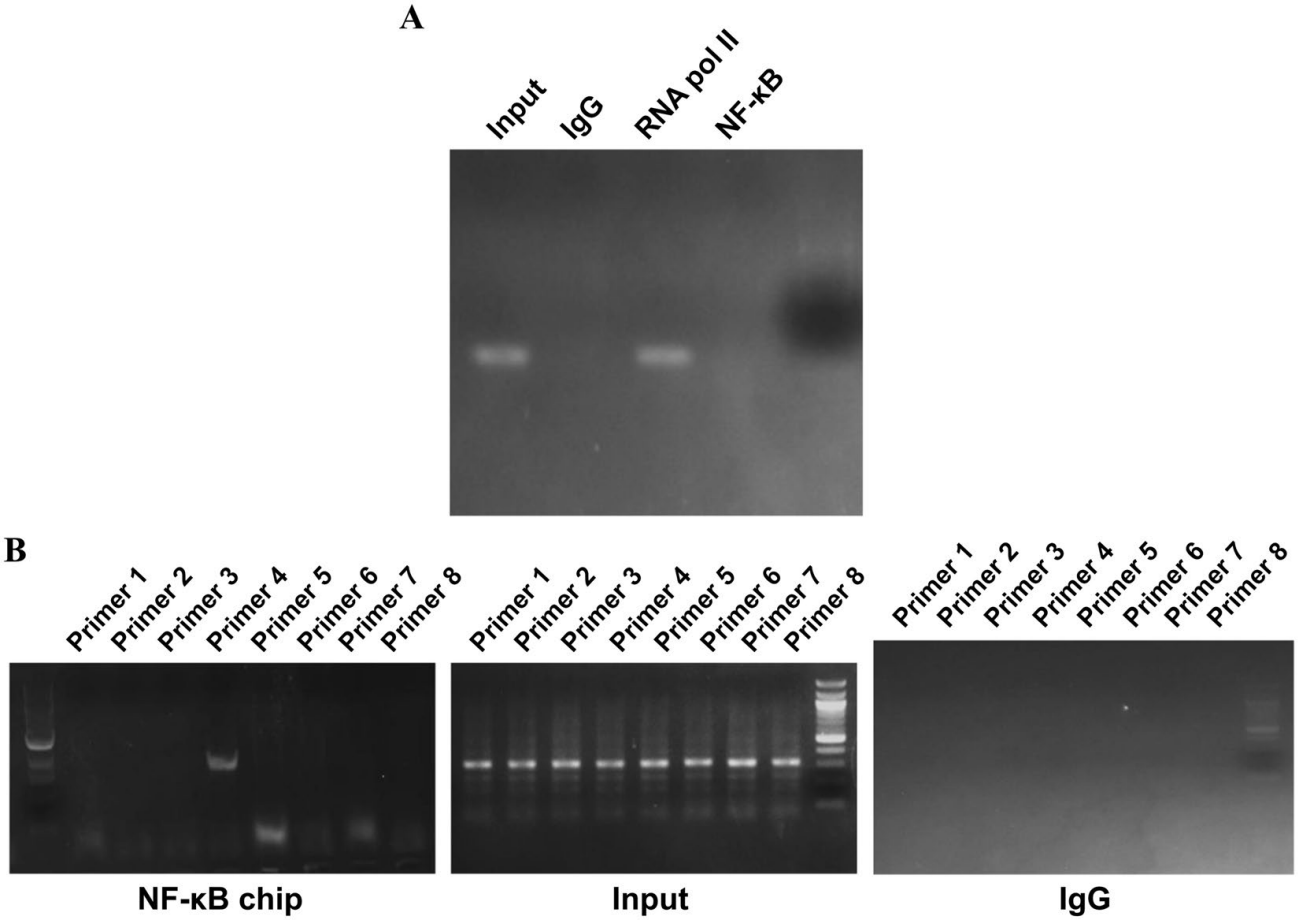
MyD88 up-regulates BDNF expression during *S. pneumoniae* meningitis by direct binding of NF-κB to the BDNF promoter. ChIP experiments were carried out in the mouse BV-2 cell strain. Nuclear extracts were analyzed by ChIP-PCR. IgG and RNA pol II antibody was used as negative and positive control. (**A**) GAPDH prime sets were performed to examine the validity. (**B**) One pair of BDNF primes amplified the ChIP products in NF-κB group.

As shown in Fig. 7, the binding site of NF-κB to the BDNF promoter was found by ChIP-PCR assay. Mouse GAPDH prime sets amplified mouse gapdh gene only in Input and RNA pol II group, examining validity of ChIP-PCR experiment (Fig. 7A). Then, one pair of primers could amplify the ChIP products to mouse bdnf gene (about 300 bp) in NF-κB group (Fig. 7B). Thus, there is a consensus NF-κB binding site present in the BDNF promoter region.

## Discussion

The present results demonstrate that *S. pneumoniae* infection drives robust gene expression of cytokines and chemokines in the CNS and peripheral immune organs, along with BDNF gene and protein expression in the cortex and hippocampus. The activity of MyD88/NF-κB signaling is not only crucial to innate immune response, but it is also required for BDNF expression. These results identify a new insight into the regulatory mechanism mediating BDNF expression during *S. pneumoniae* meningitis, thus contributing to a deep understanding of pathogenesis and pathophysiology of this disease.

*Streptococcus pneumoniae* is still the most common cause of community-acquired meningitis in developing countries^26^. Current viewpoints are that when bacteria enter the CNS, innate immune response is activated initially to limit bacteria diffusion as well as eliminate its components^27^. Although the inflammatory response can exert its defensive role, it can also induce neurotoxic effects, which are associated with cell death and neurological sequelae^8, 28^. The inefficiency of the host immune response is assumed to be associated with higher mortality of meningitis. Modulating inflammatory response and reducing side effects associated with excessive immune response have long been a hot-spot therapeutic target during bacterial meningitis. In the past decades, numerous researchers have tried different adjuvant treatments involving dexamethasone, IL-1β receptor antagonist, and mood-stabilizer lithium, among others, to improve the outcomes of *S. pneumoniae* meningitis. However, the clinical efficacy remains barely satisfactory^29–31^. Better knowledge of the pathogenesis and pathophysiology of this disease may be the sally port. Our previous results have demonstrated that BDNF increases during acute stage of *S. pneumoniae* meningitis but decreases with time^18, 32^. Similarly, BDNF concentration in the peripheral blood and injured brain increases in humans and animals suffering from trauma and ischemic insult^33, 34^. These prior studies indicated that besides its normal physiological functions, increased expression of BDNF in the CNS after a variety of insults indicates a neuro-restorative and neuroprotective role for this neurotrophic factor. Clinically, BDNF has been suggested to increase neurogenesis of neural stem cells (NSCs) and has been potentially to reduce neurological sequelae associated with meningitis and focal cerebral ischemia^32, 35^. It is estimated that there is not only the destruction of inflammatory factors but also a protective effect of neurotrophic factors. The two systems combine to the pathophysiologic process of *S. pneumoniae* meningitis.

MyD88 has been identified as TLRs adapter molecule that plays a crucial role in initiating inflammatory host immune responses to bacterial challenge mainly via TLRs engagement^28^. As it behaves dually in host immune defense, it is now widely recognized as a doubled-edged blade. MyD88-deficient mice have been demonstrated to be highly susceptible to intracerebral infection with *Escherichia coli* strain K1^36^ and have a high mortality and severe bacteremia in infectious diseases^37, 38^. In present study, we showed that absence of MyD88 decreased the resistance of mice to *S. pneumoniae* meningitis, including more weight loss and worse clinical manifestations, with diminishing neutrophil infiltration and subarachnoid hemorrhage, attenuated production of cytokines (TNF-α, IL-1β, and IL-6) and anti-inflammatory factor (IL-10) in both CNS and peripheral regions. Although significant difference in mortality between two infectious groups has not been observed in our study, there was a tendency that infected *myd88*−/− mice had a lower survival rate than infected wild-type mice. We speculate that the mortality difference would reach the level of statistical significance if we enlarge the number of animals. These results are in accordance with previous studies regarding the key role for MyD88 in immune defense but also neurons injure in both Gram-positive and Gram-negative bacterial meningitis^37, 38^. Moreover, the population of survival neurons significantly decreased and apoptosis body increased in hippocampus of infected *myd88*−/− mice compared with infected wild-type mice. Here, we also found that MyD88 underlies the ability for immune response to enhance neurotrophic expression, as *myd88*−/− mice express significantly lower BDNF production after infected with bacteria when compared with infected wild-type mice. Accordingly, this signaling adaptor molecule not only has a critical role in producing inflammatory factors during *S. pneumoniae* meningitis, but also is essential to induce neuroprotective agent. Therefore, this signal point may potentially exert the ability to protect from neuronal injury and death via balancing extent of inflammatory response and expression of neurotrophic elements.

In addition to emphasizing the ability of MyD88 in up-regulation of BDNF during *S. pneumoniae* meningitis, the present study also verified the effect of the downstream transcription factor NF-κB involved in the process. The activation of MyD88 activates downstream signal pathways, thus inducing a variety of transcription factors to translocate to the nucleus. NF-κB is one of the most major transcription factors, acting as a gate for immune response. As expected, our EMSA results confirmed previous findings that MyD88 activation increased NF-κB activation and induced p65 translocation. Potential roles for NF-κB have long been suggested in inflammation and immune response^39^ and recently in neuron survival. For example, blockade of NF-κB activity by pharmacological inhibitors attenuates CNS complications and provides protective effects for brain in bacterial meningitis^40, 41^. A recent study found that activation of NF-κB increases expression of NGF in disc cells during disc degeneration^21^. Therefore, we presume that NF-κB may be required for MyD88 to increase BDNF expression. To confirm this speculation, we performed ChIP-PCR to search for probable NF-κB binding sites with the promoter region of murine BDNF. Excitingly, this search revealed a consensus NF-κB binding site present in the BDNF promoter region, confirming a direct proof for this transcription factor in regulating BDNF. Therefore, we hypothesize that there is target point of NF-κB to both inflammation-related genes and neurotrophic elements.

In conclusion, the present findings suggested that *S. pneumoniae* meningitis induces neurotoxic cytokines and chemokines, but also neuroprotective elements. The activity of MyD88/NF-κB signaling induced innate immune response, and this pathway is also required for BDNF expression. However, how to modulate this key signal pathway to balance the effect of excessive inflammatory injury and neuroprotective survival need further investigation. BDNF undoubtedly plays a neuroprotective effect during the process of *S. pneumoniae* meningitis. Better understanding of how BDNF is regulated can be helpful to find a therapeutic target in bacterial meningitis.

## Methods

### Mouse strains and experimental design

Six-week old MyD88 knockout (*myd88*−/−) mice that have been backcrossed with C57BL/6 mice for over 10 generations, as well as wild-type littermates, were purchased from Jackson Laboratory (Bar Harbor, ME). We divided the mice into the following groups: (1) *myd88*−/− mice injected intracisternally with *S. pneumoniae* suspension (n = 12); (2) *myd88*−/− mice injected intracisternally with sterile saline (n = 8); (3) wild-type mice injected intracisternally with *S. pneumoniae* suspension (n = 12); and (4) wild-type mice injected intracisternally with sterile saline (n = 8). The animal experiments were approved by the Animal Ethical and Welfare Committee of Xinhua Hospital Affiliated to Shanghai Jiaotong University School of Medicine. All methods were performed in accordance with the relevant guidelines and regulations. We made efforts to minimize the number of animals used and their suffering.

### Infecting organisms

We used the serotype III *S. pneumoniae* strain, which was obtained from ATCC. To obtain the appropriate concentrations of bacteria, serotype III *S. pneumoniae* was cultured on a blood-agar plate for 18 h and then inoculated into Vital Aer Broth overnight at 37 °C in air with 5% CO_2_ for another 18 h to reach the logarithmic phase. The bacteria were then centrifuged and washed twice with sterile saline. We used a nephelometer to achieve a concentration of approximately 1 × 10^4^ colony forming units (cfu)/ml by re-suspending the bacteria with sterile saline.

### Animal model of meningitis

All mice (n = 40) were anesthetized with 1–1.5 ml/100 g 5% chloral hydrate by intraperitoneal injection. A 10-μl volume containing either 1 × 10^4^ cfu/ml *S. pneumoniae* or sterile saline was injected intracisternally into each mouse. At 24 h after infection with *S. pneumoniae* or saline, the health status of the mice were assessed by weighting and by a clinical score: 0 = no apparent behavioral abnormality; 1 = moderate lethargy (apparent decrease of spontaneous activity); 2 = severe lethargy (rare spontaneous movements, but walking after stimulation by the investigator); 3 = unable to walk; and 4 = dead^42^. After clinical evaluation, mice from all groups were sacrificed at 24 h after inoculation to harvest brains and spleens. In order to document the development of bacterial meningitis, cerebellum homogenates were plated and cultured on blood-agar plates.

### Histopathological evaluation of murine brain sections

At 24 h after inoculation, all surviving animals were anesthetized and perfused through the left ventricular with 50 ml normal saline. Then, the animals were decapitated, the brains were removed, followed by segmentation of the brains into 2 hemispheres. The left hemispheres were fixed in 4% paraformaldehyde overnight at 4 °C, embedded in paraffin, and cut into 4-μm-thick coronal sections through the hippocampus. The sections were stained with hematoxylin & eosin (H&E) (Beyotime Biotechnology, China) and cresyl violet (Sigma-Aldrich, USA) according to a standard protocol. Apoptosis-like cell death was determined by terminal deoxynucleotidyl transferase dUTP nick end labeling (TUNEL) immunofluorescence staining using the *In-Situ* Cell Death Detection Kit (Roche, USA) according to the manufacturer’s instructions.

### Quantification of cytokine and chemokine secretion

Brains and spleens were sonicated in PBS buffer containing a proteinase inhibitor cocktail and centrifuged at 12000 g for 15 min to remove cellular debris. The concentrations of TNF-α, IL-1β, IL-6, and IL-10 in supernatant were determined using commercial enzyme-linked immunosorbent assay (ELISA) kits (eBioscience, USA). Cytokine and chemokine levels in brain homogenates were normalized to total brain weight and reported as picograms per milliliter.

### Electrophoretic mobility shift assay for NF-κB activation

Nuclear extract preparation and electrophoretic mobility shift assay (EMSA) were performed using the EMSA kit (Thermofisher, USA) as described previously, with some modifications^34^. Briefly, nuclear extract (20 ug) was incubated with binding buffer and nonspecific oligonucleotides for 15 min, and then incubated with NF-κB biotin-labeled oligonucleotide probes (forward: 5′-AGT TGA GGG GAC TTT CCC AGG C-3′; reverse: 5′-G CCT GGG AAA GTC CCC TCA ACT-3′) for another 15 min. For super shift assays, NF-κB antibody was added along with the binding buffer. Subsequently, samples were separated by electrophoresis in a 5.5% polyacrylamide gel with 0.25 × Tris-borate-EDTA buffer. The retarded bands were detected by chemiluminescence.

### Analyses of BDNF at transcriptional and translational level

Reverse transcription polymerase chain reaction (RT-PCR) was performed as previously described^43^. Briefly, total RNA from brain tissues (cerebral cortex and hippocampus) were extracted using Trizol reagent (TaKaRa, Japan) and isolated using chloroform following the manufacturer’s instructions. RNA was converted to cDNA by using the Primer-Script One-Step RT-PCR kit (TaKaRa). Real-time PCR was performed using the ABI7500 system (Applied Biosystems, Carlsbad, CA) using the SYBR Premix Dimmer Eraser kit (TaKaRa) to amplify the cDNA template. The forward and reverse strand primers that were used to amplify the mRNA encoding mouse BDNF were ATTAGCGAGTGGGTCACAGC and TCAGTTGGCCTTTGGATACC, respectively. The primers were synthesized by Shanghai Sangon Biological Engineering Technology Company Limited. BDNF gene expression in each sample was normalized to β-actin expression. The relative expression level of mRNAs was calculated by the 2^−ΔΔCt^ method.

To assay BDNF synthesis, BDNF ELISA kit (Cusabio, China) was used to quantify protein concentration in cerebral cortex and hippocampus according to the manufacturer’s instruction.

### Chromatin immunoprecipitation assay

Chromatin immunoprecipitation (ChIP) were performed with the BV-2 cell strain (a standard microglia cell stain). Cellular protein-DNA complexes were cross-linked, isolated and fragmented. Immunoprecipitation was performed with NF-κB antibody (Millipore, USA) to enrich specific chromatin fragments. RNA pol II antibody (Santa Cruz, USA) was added as positive control, and goat IgG was used as negative control. Antibody complexes were collected and washed, then reversed the cross-links. The amounts of immunoprecipitated DNA were calculated by comparison to input DNA. The ChIP products were also analyzed by PCR. The primer sets used to amplify mouse *bdnf* gene are listed in supplementary data.

### Statistical analysis

The Shapiro-Wilk test and Levene test were used to distinguish between parametric and nonparametric values. Differences among groups were analyzed using two-way ANOVA (MyD88 and meningitis) for parametric data, followed by Tukey’s post-hoc test; otherwise, the Mann-Whitney test was used, the intergroup comparisons were performed using Wilcoxon’s tests. If not stated otherwise, the data was reported as the mean ± SD. Survival rates were compared by the log rank test. Differences were considered significant at p < 0.05. All graphs were generated using Graph Pad Prism 5.0. Statistical analyses were performed using SPSS software version 18.0.

## Electronic supplementary material

supplementary data
