# Supplementary material for: Brain-derived neurotrophic factor is regulated via MyD88/NF-κB signaling in experimental Streptococcus pneumoniae meningitis
Source: Sci Rep. 2017 Jun 14;7:3545. doi: 10.1038/s41598-017-03861-z (PMC5471242; doi:10.1038/s41598-017-03861-z)
Supplement: Supplementary file 1 — supplementary data [file 41598_2017_3861_MOESM1_ESM.pdf]

Brain-derived neurotrophic factor is regulated via MyD88/NF-κB signaling in experimental *Streptococcus pneumoniae* meningitis

Running title: MyD88/NF-κB regulates BDNF in meningitis

Danfeng Xu[a], Di Lian[a], Zhijie Zhang[a], Ying Liu[b], Jiaming Sun[c], Ling Li[a, *]

[a] Department of Pediatric Neurology, Xinhua Hospital Affiliated to Shanghai Jiaotong University School of Medicine, Shanghai 200092, PR China

[b] Department of Clinical Laboratory, Xinhua Hospital Affiliated to Shanghai Jiaotong University School of Medicine, Shanghai 200092, PR China

[c] Department of Pathology, Xinhua Hospital Affiliated to Shanghai Jiaotong University School of Medicine, Shanghai 200092, PR China

Supplementary data The primer sets used to amplify mouse *bdnf* gene

Gene	Forward primer sequence (5'-3')	Reverse primer sequence (5'-3')
1	GTAAATGCTTACAAAATCGA	CAAAGCTAGCCTATCCTACA
2	TGTAGGATAGGCTAGCTTTG	AAGAACTGCTCGGACCACAT
3	ATGTGGTCCGAGCAGTTCTT	ACTAGTGCAGCATCTCTCTG
4	CAGAGAGATGCTGCACTAGT	TCCACCAGAAGGAAGCATGT
5	ACATGCTTCCTTCTGGTGGA	TGAGTAGTACTTTACACGCT
6	AGCGTGTAAAGTACTACTCA	CCAACGTTTTACTACATCTG
7	CAGATGTAGTAAAACGTTGG	GGTTTAATTTTTAATGATCT
8	AGATCATTAAAAATTAAACC	AACTGTGGGAAGGAAGCAGA
